# Analysis of Association of Genetic Markers in the LUZP2 and FBXO40 Genes with the Normal Variability in Cognitive Performance in the Elderly

**DOI:** 10.1155/2018/2686045

**Published:** 2018-04-19

**Authors:** Vadim Stepanov, Kseniya Vagaitseva, Anna Bocharova, Andrey Marusin, Valentina Markova, Larisa Minaycheva, Oksana Makeeva

**Affiliations:** ^1^Institute of Medical Genetics, Tomsk National Medical Research Center, Tomsk 634050, Russia; ^2^Tomsk State University, Tomsk 634050, Russia; ^3^Nebbiolo Center for Clinical Trials, Tomsk 634009, Russia

## Abstract

Cognitive performance is an important endophenotype for various neurodegenerative and neuropsychiatric traits. In the present study two genetic variants in the leucine-zipper protein (LUZP2) and the F-box 40 protein (FBXO40) genes, previously reported to be genome-wide significant for Alzheimer's diseases and schizophrenia, were examined for an association with cognitive abilities in normal elderly from the Russian population. Rs1021261 in the LUZP2 and rs3772130 in the FBXO40 were genotyped by multiplex PCR and MALDI-TOF mass spectrometry in a sample of 708 normal elderly subjects tested for cognitive performance using the Montreal Cognitive Assessment (MoCA). Association of genetic variability with the MoCA scores was estimated by parametric and nonparametric analysis of variance and by the frequency comparison between upper and lower quartiles of MoCA distribution. Significantly higher frequency of “TT” genotype of rs1021261 in the LUZP2 gene as well as “A” allele and “AA” genotype of rs3772130 in the FBXO40 gene was found in a subsample of individuals with the MoCA score less than 20 comparing to the fourth quartile's subsample (MoCA > 25). The data of the present study suggests that genetic variability in the LUZP2 and FBXO40 loci associated with neurodegenerative and neuropsychiatric diseases is also contributed to the normal variability in cognitive performance in the elderly.

## 1. Introduction

Cognitive decline with the age, both in the normal aging and in the pathological manifestations in form of dementia, is an important public health and social challenge. Individual variability of cognitive functions is an important endophenotype of many neurodegenerative, psychiatric, and mental diseases, such as schizophrenia (SZ) [1], Alzheimer's diseases (AD) [2], bipolar disorder [3], and attention-deficit hyperactivity disorder [4]. Recent genome-wide association studies (GWAS) have revealed dozens of single nucleotide polymorphisms (SNPs) associated with a cognitive performance in SZ or AD patients, as well as in normal healthy subjects [5–10]. Some of genome-wide significant cognitive functions markers demonstrate an association with diseases as well, indicating overlapping genetic mechanisms underlying normal and pathological neurocognitive traits. Among genome-wide significant AD markers, revealed in the recent GWA studies, there are genetic variants in the leucine-zipper protein 2 (LUZP2) and the F-box 40 protein (FBXO40) genes.

LUZP2 gene on chromosome 11 encodes a leucine-zipper protein of unknown function, which is normally expressed only in the brain and the spinal cord. LUZP2 gene is deleted in some patients with Wilms tumor, aniridia, genitourinary anomalies, and mental retardation (WAGR) syndrome [11]. Polymorphic variants in this gene were reported to be associated with the late-onset Alzheimer's disease [12], as well as with schizophrenia [13, 14], intelligence [15], and verbal memory [16]. Also a duplication which includes the LUZP2 gene was observed in AD patient [17]. Haplotypes around intronic rs1021261 in the LUZP2 gene were genome-wide significantly associated with the intelligence in an ancestrally homogeneous family sample of individuals with at least one child affected by attention-deficit hyperactivity disorder (ADHD) [15].

FBXO40 gene on chromosome 3 encodes a member of the F-box protein family which is characterized by an approximately 40-amino acid F-box motif. F-box 40 protein is substrate-recognition component of the SCF (SKP1-CUL1-F-box protein)-type E3 ubiquitin ligase complex that may function in myogenesis and in insulin growth factor (IGF) signaling in the brain and CNS. Potentially FXBO40 may be involved in neurodegenerative and neuropsychiatric diseases through alterations of the IGF-I neuronal modulations. Genetic variants in the FBXO40 gene were found genome-wide significant for Alzheimer's disease in the APOE e4 carriers [18] and skin aging [19]. Intronic variant rs3772130 in the FBXO40 gene was reported as associated with a cognitive performance in GWAS [6].

Thus genetic variability in LUZP2 and FBXO40 genes may contribute to neurodegenerative and neuropsychiatric diseases as well as to normal cognitive phenotypes. This study aimed to examine whether the rs1021261 in the LUZP2 gene and rs3772130 in the FBXO40 gene are associated with cognitive performance in the normal elderly population.

## 2. Material and Methods

### 2.1. Subjects

A sample of 708 elderly subjects (age range between 59–89 years, the mean age 70.8 years) of Russian descent was randomly selected from a population-based cohort study on primary prevention of the Alzheimer's disease in Tomsk, Russia [20, 21]. Cognitive performance was assessed using the Montreal Cognitive Assessment (MoCA) as previously described in [20]. MoCA measures 8 cognitive domains including memory, attention, naming, visuospatial/executive, language, abstraction, delayed recall, and orientation. MoCA scores ranged between 0 and 30 points, and the higher scores indicate the better cognitive performance [22]. The MoCA has been translated into many languages including Russian (see https://www.moca.org/). In this study official Russian version of the MoCA was used [23]. All subject had Russian as primary spoken language. The study was approved by the Ethics Review Board at the Institute of Medical Genetics, Tomsk, Russian Federation.

### 2.2. Genotyping

**Figure 1:**
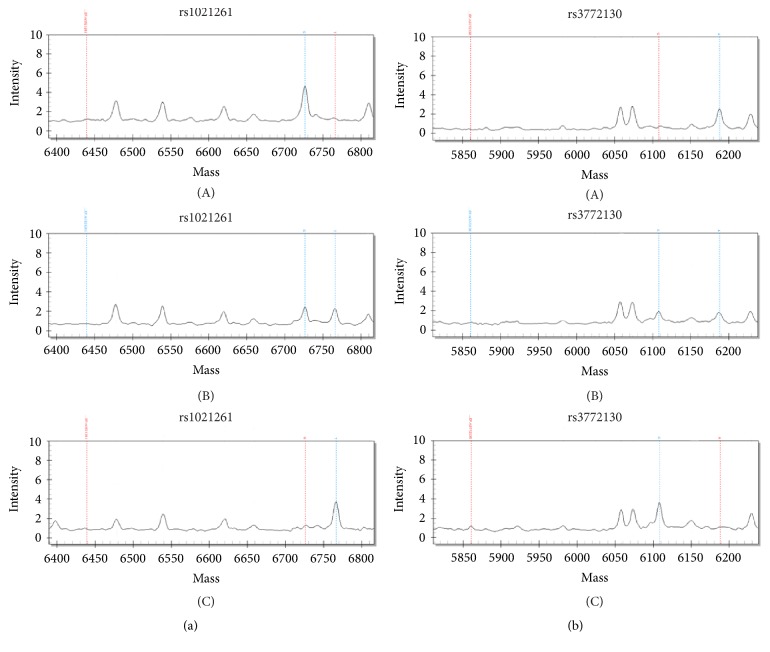
Single nucleotide polymorphism genotyping by MALDI-TOF mass spectrometry on Sequenom MassARRAY 4 platform. Fragments of mass spectra of specimens with different genotype. (a) Rs1021261: GG (A), GT (B), and TT (C). (b) Rs3772130: AA (A), AG (B), and GG (C).

Intronic single nucleotide polymorphic variant (SNP) rs1021261 in the LUZP2 gene resulted from the G-T transversion in the position 24660211 (human genome build GRCh38.p7) on chromosome 11. Intronic rs3772130 in the FBXO40 gene results in a substitution of A to G in the position 121625293 (GRCh38.p7) of chromosome 3. Genotyping of the two SNPs was performed by multiplex PCR with the following iPLEX primer extension reaction and detection of allele-specific extension products by matrix-assisted laser desorption/ionization time-of-flight (MALDI-TOF) mass spectrometry on Sequenom MassARRAY 4 platform. Primers (Table 1) were designed using the Sequenom Assay Design software (Agena Bioscience) available online at https://www.sequenom.com. Details of the genotyping method have been previously described elsewhere [24]. Briefly, genomic regions around the polymorphic positions were amplified by PCR and then SAP reaction was used to dephosphorylate unincorporated dNTPs with alkaline phosphatase (SAP). On the next step the PCR products were incubated with the extension primers (iPLEX primers) adjacent to the polymorphic position. Extension of the iPLEX primer by incorporating modified nucleotides found in the polymorphic position was followed by the mass-spectrometry separation of extended DNA molecules. The real-time analysis of mass spectra was performed with the MassARRAY TYPER 4.0 software (Agena Bioscience).  Figure 1 represents examples of the mass spectra for specimens with different rs112183431 and rs3772130 genotypes.

### 2.3. Statistical Analysis

We used two approaches to examine whether genetic variation in LUZP2 and FBXO40 genes is associated with cognitive performance in the elderly population. First, total values of individual MoCA scores were treated as quantitative trait, and associations of MoCA values with genetic variability were tested using parametric (analysis of variance, ANOVA) and nonparametric (Kruskal-Wallis test and median test) statistics implemented in the Statistica 7.0 software (StatSoft Inc.). For ANOVA analysis MoCA scores were adjusted for age and education using linear regression model. Second, the total sample was subdivided into quartiles of MoCA distribution, and differences in allele and genotype frequencies between lower and upper quartiles were estimated in case-control analysis by Fisher's exact test.

## 3. Results

### 3.1. Alleles and Genotypes Frequency

Alleles and genotypes frequency of polymorphic variants in the LUZP2 and FBXO40 genes in a sample of 708 elderly subjects from the Russian population as well as in the first (MoCA < 20) and the fourth (MoCA > 25) quartiles of MoCA scores distribution are presented in the Table 2. Both polymorphic variants are quite common in populations of European origin. Frequency of the minor allele “T” of the rs1021261 in LUZP2 in the total sample (0.309) and the minor allele “G” of the rs3772130 in FBXO40 (0.255) fall within the frequency range observed in populations of the European descent in the 1000 Genomes project [https://www.ncbi.nlm.nih.gov/variation/tools/1000genomes/].

### 3.2. Comparison of Lower and Upper Quartiles of MoCA Scores

Significantly higher frequency of the rs1021261 “TT” genotype in the LUZP2 gene as well as the rs3772130 “A” allele and “AA” genotype in the FBXO40 gene was found in the subsample of individuals with the MoCA score less than 20 comparing to the fourth quartile's subsample (see Table 2). Odds ratio values for the lower MoCA score associated with “A” allele and “AA” genotype of the rs3772130 were 1.55 (95% CI 1.03–2.33, *p* = 0.029) and 1.86 (95% CI 1.12–3.10, *p* = 0.014), respectively. Odds ratio for the lower MoCA for “TT” genotype of the rs1021261 was 4.52 (95% CI 1.01–5.37, *p* = 0.043).

### 3.3. Parametric and Nonparametric Tests for MoCA Score Differences among Genotypes

Mean values of MoCA scores in the subjects with different genotypes in the total sample of 708 elderly and the analysis of variance results are presented in Table 3. One-way ANOVA demonstrates differences in the mean MoCA scores among genotypes on the margin of significance for both genetic variants (*p* = 0.065 for LUZP2 and *p* = 0.069 for FBXO40). Nonparametric median test shows similar results for the LUZP2 genetic variant (chi-square = 5.17, df = 2, *p* = 0.075), while effect of genotypes of the FBXO40 gene on the MoCA scores proved to be significant (chi-square = 8.26, df = 2, *p* = 0.016).

## 4. Discussion

Genetic bases of cognitive performance in normal aging and dementias are the subject of intensive research. Many common genetic variants, including dozens of SNPs and CNVs in genes of unknown relations to CNS and the brain functions, have been reported in GWAS to be contributed to cognitive performance in patients with the Alzheimer's diseases and other neurodegenerative disorders, as well as in the healthy subjects [5–10] (see also GWAS catalog at https://www.ebi.ac.uk/gwas/). Whole exome sequencing studies demonstrate that a rare genetic variation also may play a substantial role in the heritable component of normal and impaired cognitive abilities, as well as in the general intelligence [25–27].

Despite being controversial, literature data indicate that common genetic variation in the LUZP2 and FBXO40 genes may contribute to neurodegenerative and neurocognitive phenotypes. In the present study we examined the association of two intronic variants in LUZP2 and FBXO40, previously reported genome-wide significant for the late-onset Alzheimer's disease, with the cognitive performance in a normal elderly population. Both variants demonstrate marginal *p* values in the analysis of between-genotypes variance in MoCA scores in the total sample of elderly subjects, but their allele and/or genotype frequency differs significantly between the subjects with highest and lowest MoCA scores. Considering population variability in normal cognitive performance and memory functions as the endophenotype for Alzheimer's and dementia, one might suggest that neurocognitive functions in norm and in neurodegenerative diseases have overlapping genetic background and, consequently, an overlapping pattern of genetic associations.

In the previous study the minor allele G of the rs3772130 in FBXO40 gene has been found to be associated with the higher performance in the Cambridge Neuropsychological Test Automated Battery (CANTAB) [6]. Our results confirm the lower frequency of the major allele A and homozygous genotypes AA in the subjects with lower cognitive performance. As reported by Loo et al. [15], rare haplotypes around rs1021261 in LUZP2 gene were associated with the higher intelligence in an ancestrally homogeneous family sample of individuals with at least one child affected by attention-deficit hyperactivity disorder (ADHD), while in our study the rare homozygous genotype was significantly more common in the lower quartile of the MoCA distribution.

No direct functional evidence of the involvement of LUZP2 or FBXO40 proteins in neurodegenerative process in AD and dementia has been found. However the potential role of FBXO40 in the neurodegeneration may be associated with the modulation of insulin growth factor (IGF) signaling in the brain via participation of the Fbxo-40 protein in ligase complexes involved in the degradation of insulin receptor substrates. IGF-I is highly expressed within the brain and is essential for normal brain development [28]. IGF-I enhances nerve cell metabolism and modulates neuronal excitability, two properties that are crucial for the ability of IGF-I to protect neurons against insults [29, 30]. At the tissue level, IGF-I stimulates angiogenesis, regulates amyloid load, and modulates the activity of neuronal circuitries [31–33]. Dysregulation of IGF signaling was reported to be involved in many types of age-related diseases including neurodegenerative disease such as late-onset Alzheimer's disease and familial Parkinson's diseases along with the diabetes, cancer, arteriosclerosis, and osteoporosis [31, 34–36]. Recently it has been discovered that insulin receptor substrates (IRS) involved in IGF/insulin signaling form the high-molecular-mass complexes (IRSomes), which are the major substrates of receptor kinases, mediating IGF/insulin signals to direct bioactivities [37]. IFG/insulin signaling is directed by the ubiquitination and degradation of IRSs mediated by E3 ligase [38, 39]. Several E3 ligases have been reported to induce the degradation such as cullin7- (CUL7-) Fbxw8 E3 ligase complex and SCF (Skp1, cullin1, and Rbx1)-Fbxo40 E3 ligase complex. Knockdown of Fbxo40 in mice also increased IRS protein levels and activation of downstream pathways [40]. The proposed mechanism of the phenotypic impact of the intronic variant in the FBXO40 gene might be also related to the possible transcription regulation effects of the rs3772130. Compilation of the data obtained by the Genotype-Tissue Expression (GTEX) Consortium, Roadmap Epigenomics Consortium [41], and genome-wide and meta-analysis studies of expression quantitative trait loci (eQTL) [42, 43] indicate that rs3772130 may serve as cis- and trans-eQTL involved in the regulation of expression of FBXO40 and IQCB1 genes in the variety of tissues including brain.

Plausible role of the LUZP2 in neurocognitive functions may be related to its impact on neuroendocrine differentiation. Luzp2, a leucine-zipper motif containing transcription factor, is highly expressed in the brain and spinal cord [11] and represents the positive modulator of neuroendocrine differentiation [44]. The CNS-specific expression pattern of luzp2 is possibly explained by upstream neuron-specific enhancer [45]. Inoue et al. [46] recently have found that expression of Luzp2 in the upper-layer neocortical neurons is significantly downregulated in mice homozygous for knockout of Prdm8, a member of the PR domain protein family, specifically expressed in the postnatal upper-layer neocortex. Accumulating evidence of the LUZP2 role in the genetic component of a spectrum of neurocognitive phenotypes, based on genome-wide association with AD, schizophrenia, cognitive, and memory functions [11–17], as well as on the findings of exonic variants shared among the affected siblings with a familial variant of Alzheimer's disease with neuroimaging features of Alzheimer's disease but lacking amyloid-b deposits in the brain [47], suggests its functional implication in the neurodegeneration and impaired cognition in humans.

## 5. Conclusion

In summary, in the present study we observed the association with cognitive performance estimated by MoCA for genetic variants in the LUZP2 and FBXO40 genes previously linked to the AD and schizophrenia in GWA studies. The data of the present study indicate that genetic variability contributed to neurodegenerative and neuropsychiatric diseases may be expressed in norm or in preclinical stages as markers of cognitive functions.

## Figures and Tables

**Table 1:** PCR and iPLEX primers used for genotyping of rs1021261 and rs3772130 by multiplex PCR and MALDI-TOF mass spectrometry.

Primer	Sequence (5′-3′)
LUZP2 rs1021261	
PCR forward	ACGTTGGATGTTCCCACAAAGGATTTGCAG
PCR reverse	ACGTTGGATGCTGAAAGAATTTGTGTGAGAC
iPLEX extension	CTCCTCATTCAGGGAAGAAAG

FBXO40 rs3772130	
PCR forward	ACGTTGGATGTCTCATGGTAAACCTGTTGG
PCR reverse	ACGTTGGATGGGAAGAAATTCAACAGTGAG
iPLEX extension	AGATTGACCTAAATGGGCA

**Table 2:** Allele and genotype frequency of two genetic variants in LUZP2 and FBXO40 genes in the total sample and in the lower (Q1) and upper (Q4) quartiles of the MoCA distribution.

Allele, haplotype	Total sample, *N* = 708	Lower quartile (MoCA < 20), *N* = 152	Upper quartile (MoCA > 25), *N* = 132	Q1 versus Q4, *p*
		LUZP2 rs1021261		
G	0.691	0.635	0.667	0.480
T	0.309	0.365	0.333	
GG	0.383	0.428	0.409	0.809
GT	0.480	0.414	0.515	0.096
TT	0.137	0.158	0.076	**0.043**

		FBXO40 rs3772130		
A	0.745	0.799	0.720	**0.029**
G	0.255	0.201	0.280	
AA	0.572	0.671	0.523	**0.014**
AG	0.347	0.257	0.394	**0.015**
GG	0.081	0.072	0.083	0.825

**Table 3:** One-way ANOVA analysis of MoCA scores among genotypes of genetic variants in LUZP2 and FBXO40 genes.

Genotype	*N*	MoCA mean	MoCA std. deviation
	LUZP2 rs1021261, *F* = 2.731, *p* = 0.065		
GG	268	21.978	4.002
GT	336	22.414	3.759
TT	96	21.406	4.113
All	674	22.106	3.913

	FBXO40 rs3772130, *F* = 2.681, *p* = 0.069		
AA	401	21.843	3.993
AG	243	22.572	3.766
GG	57	21.965	3.831
All	701	22.106	3.913
